# Twelve year experience of laparoscopic gastric plication in morbid obesity: development of the technique and patient outcomes

**DOI:** 10.1186/1750-1164-6-7

**Published:** 2012-08-22

**Authors:** Mohammad Talebpour, Seyed Mohammad Kalantar Motamedi, Atieh Talebpour, Hamed Vahidi

**Affiliations:** 1 Laparoscopic Surgical Ward, Sina Hospital, Tehran University of Medical Sciences, Tehran, Iran; 2 Endocrinology and Metabolism Research Center, Tehran University of Medical Sciences, Tehran, Iran

**Keywords:** Morbid obesity, Laparoscopy, Gastric plication, Restriction

## Abstract

**Background:**

Laparoscopic Gastric Plication (LGP) is a new restrictive bariatric surgery, previously introduced by the author. The aim of this study is to explain the modifications and to present the 12-year experience, regarding early and long term results, complications and cost.

**Methods:**

We used LGP for morbid obesity during the past 12 years. Anterior plication (10 cases), one-row bilateral plication while right gastroepiploic artery included (42 cases), and excluded from the plication (104 cases) and two-row plication (644 cases). The gastric greater curvature was plicated using 2/0 prolen from fundus at the level of diaphragm preserving the His angle to just proximal to the pylorus. The anatomic and functional volume of stomach was 50cc and 25cc respectively in two-row method. Ordered postop visits also included evaluation of weight loss, complications, change of diet and control of exercise.

**Results:**

LGP was performed in 800 cases (mean age: 27.5, range: 12 to 65 years, nine under 18). Female to male ratio was 81% to 19% and average BMI was 42.1 (35-59). The mean excess weight loss (EWL) was 70% (40% to 100%) after 24 months and 55% (28% to 100%) after 5 years following surgery. 134 cases (16.7%) did not completed long term follow-up. The average time of follow up was 5 years (1 month to 12 years). 5.5% and 31% of cases complained from weight regain respectively during 4 and 12 years after LGP. The mean time of operation was 72 (49–152) minutes and average hospitalization time was 72 hours (24 hours to 45 days). The cost of operation was 2000 $ less than gastric banding or sleeve and 2500 $ less than gastric bypass. Eight patients out of 800 cases (1%) required reoperation due to complications like: micro perforation, obstruction and vomiting following adhesion of His angle. Other complications included hepatitis pneumonia, self-limiting intra-abdominal bleeding and hypocalcaemia.

**Conclusion:**

The percentage of EWL in this technique is comparable to other restrictive methods. The technique is safe with 1.6% complication (1% reoperated), and 31% regain during 12 years. The cost of operation is less than the other methods.

## Background

Modern life has influenced on amount of physical activity and form of diet [1-4]. As a result there is an increasing trend in prevalence of morbid obesity around the world [5-8]. Although there are few cases with congenital, genetic or hormonal etiology of morbid obesity, the main factor is habitual. Change of lifestyle including more exercise and a low fat and calorie diet is the fundamental plan for morbid obese patients treatment [9].

Influence of diet and exercise in morbid obese patients is about 10% in long term period; thus, in case of lifestyle modification failure, bariatric surgery could be considered [10]. They need a potential trigger for weight loss like restrictive bariatric surgeries that are effective to preserve diet for about 4 years [11,12]. Other methods have limited and temporary effect. Such methods are not recommended choices in long term i.e. drugs and hypnosis etc. [13].

Restrictive methods (RM) are the most conservative bariatric surgery especially for newly risen type of morbid obese patients as they are more knowledgeable nowadays. This group mainly contains patients with history of high calorie intake and low activity during adolescence. But patient has a new insight to lose weight due to maturity of mind and finding problems of obesity after puberty.

The mean EWL in different forms of restrictive methods is almost the same [14-17]. Efficacy of RM is not mainly related to the type of technique. Although adequate weight loss always occurs, patient’s cooperation is the key factor for its effectiveness. Long term results showed that weight regain only occurs in a few cases due to temporary effect of restrictive methods and discontinuing diet and exercise [18,19]. Malabsorptive method has longer effect on weight loss but the risk of late complications due to vitamin deficiency and anemia is noticeable [20].

As the number of these patients is increasing, and also different restrictive methods cause relatively considerable rate of early complications [21-25], author designed a new restrictive method after different stages of animal study named as Laparoscopic Gastric Plication (LGP) 12 years ago [26,27]. The main aim of this paper is to present the 12 year experience on more than 800 cases of LGP and their long term outcome data. As this technique has been improved as a result of study on Iranian cases, and it has gained currently widespread usage in the world, the author chose the *Iranian Method* title on the honor of these patients.

## Methods

This prospective case series study has been mainly performed in Laleh and Sina Hospitals, Tehran, Iran by the first author since 12 years ago.

### Patients

After some years of experience, based on wide range of EWL results and assessment of individuals, the key factor for case selection was related to one’s motivation and cooperation. Gastric plication was selected for cases with potential for continuous diet and exercise after operation. They are mostly young single females with the history of obesity during adolescence and hate of obesity at the time of operation. In cases with moderate rate of motivation the preferred method was mixed technique (gastric bypass). They were mainly males, diabetic cases, and middle aged females. The third group with poor motivation and predominantly psychological problems was candidate for malabsorptive technique without any restrictive option (Table 1).

**Table 1:** Indication of bariatric surgery

**METHOD/LANDMARK**	**MOTIVATION**	**SAMPLE**
** LGP**	EXCELLENT	YOUNG FEMALE
** GBP**	MODERATE	MALE DIABETIC
** DS**	POOR	SUPER OBESE

*LGP*: Laparoscopic gastric plication, *GBP*: Gastric bypass, *DS*: Duodenal switch.

Any patient with BMI over 40 or 35 with comorbidity was selected for LGP. During the first 6 years of experience only patients with age of more than 18 years were selected. But afterwards when LGP method was modified, this was also applied for some selected well oriented adolescent patients. LGP performed for adolescents after psychological consult and achieving good motivation.

If this operation made acceptable excess weight loss (EWL) even in super obese cases, there was no need for second stage operation. But in weight regain cases (who had appropriate primary progress in weight loss during first year but their trend reversed into weight gain afterwards; so that their current cumulative weight loss is less that 30% of primary excess weight) or patients with unacceptable EWL (less than 30% after 6 months), the second stage operation was advised after 4 years in regain and 6 months in failure cases. At this stage without any change in the first part, laparoscopic malabsorptive method was performed. In patients who had complained about GERD as a comorbidity of morbid obesity, the plan of operation was funduplication of Nissen and plication of the remaining of stomach. If hiatal hernia was seen during operation closure of crus was enough.

Characteristic data was taken using standard questionnaire and patients were interviewed to evaluate their psychological issues and motivation. Anthropometric measures were recorded and informing consent was taken. Preop work ups included sonography of gallbladder, hormonal, electrolytes, liver function and blood coagulation tests.

### Setup

**Figure 1:**
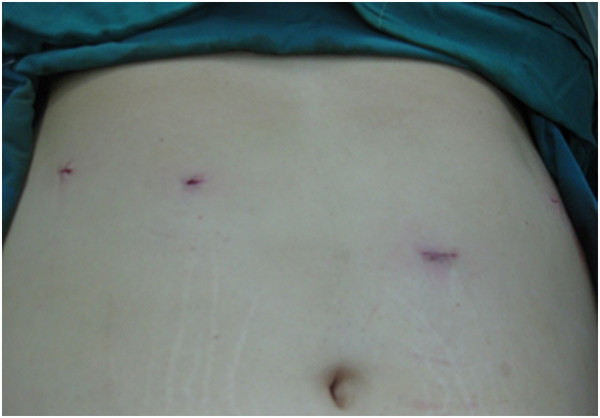
Trocar position of LGP.

Lower limb bandage, prophylactic heparin, pantazol and antibiotic therapy were advised in all cases. Position of patients was supine with 30-degree reverse trendelenburg. Pneumoperitoneum performed by CO2 with 12-15 mmHg pressure via left subcostal insertion of Verress needle at mid-axillary line. The first trocar placed at left paramedian line 20 cm away from xyphoid angle (telescope trocar). Left and right hands of surgeon’s trocars were inserted based on ergonomic assessment at this stage (left middle axillary line at subcostal (insertion site of Verress needle) and right mid-clavicular line at 5 cm above the first trocar). The second surgeon’s trocar was inserted at right anterior axillary line. Almost always one 10 mm and three 5 mm trocars used (Figure 1).

### Dissection

Anterior wall of stomach at prepyloric area grasped and pulled up at first step. Dissection was started in contact to gastric wall releasing the greater curvature from prepyloric area up to almost 2 cm to His angle, (just after separation of stomach from spleen in contact to diaphragm) preserving the anatomy of His angle and sacrificing left and right gastroepiploic artery. All greater curvature vessels were ligated by intra corporeal suturing, clips or coagulation at earlier times (2000 to 2004) and by LigaSure^TM^ or Ultracision later on.

### Plication

**Figure 2:**
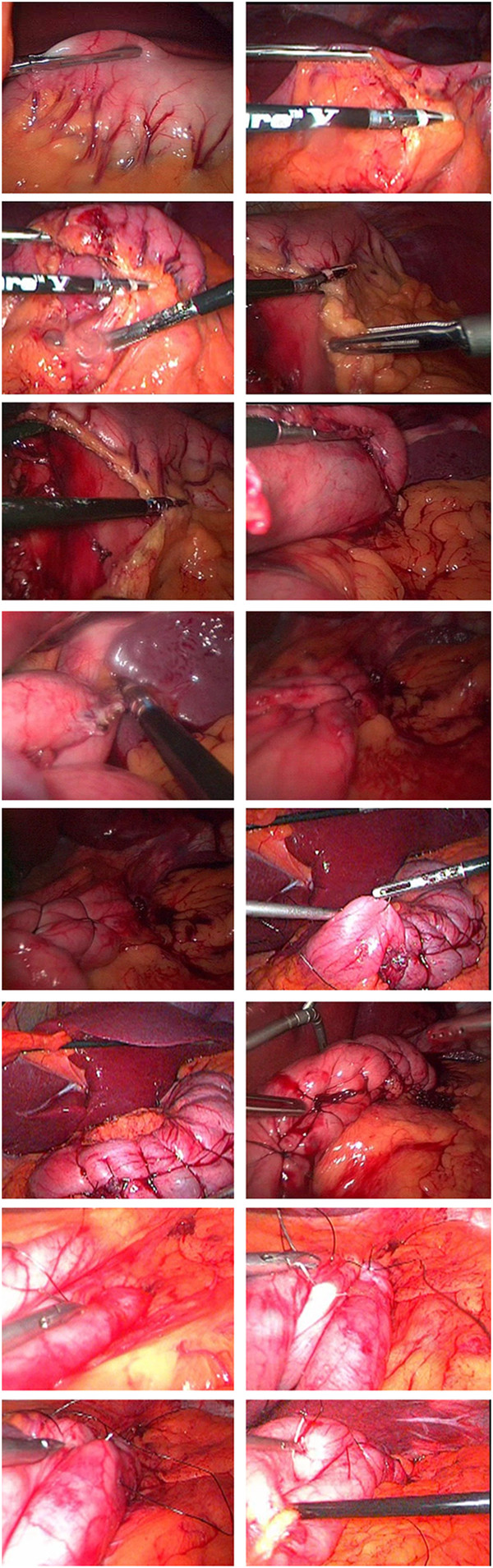
Different steps of LGP.

**Figure 3:**
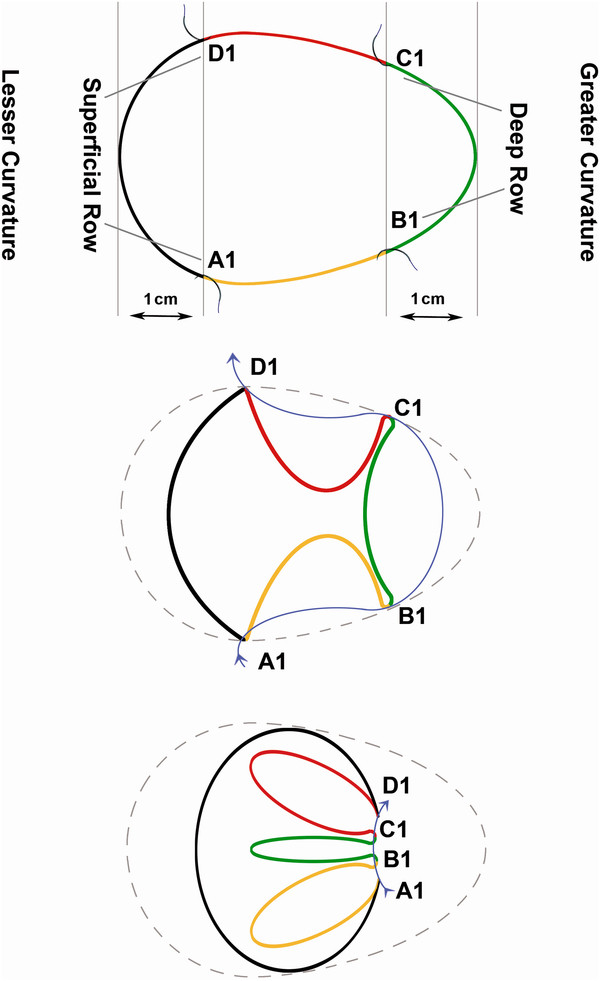
Transverse section of plicated stomach.

**Figure 4:**
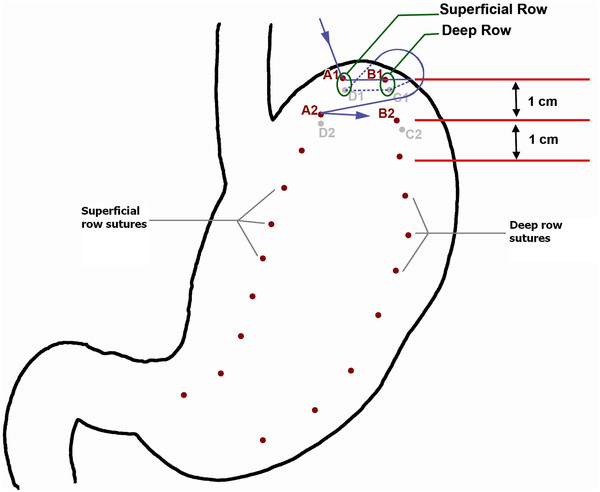
Anterior view before plication with marked points of each bite.

**Figure 5:**
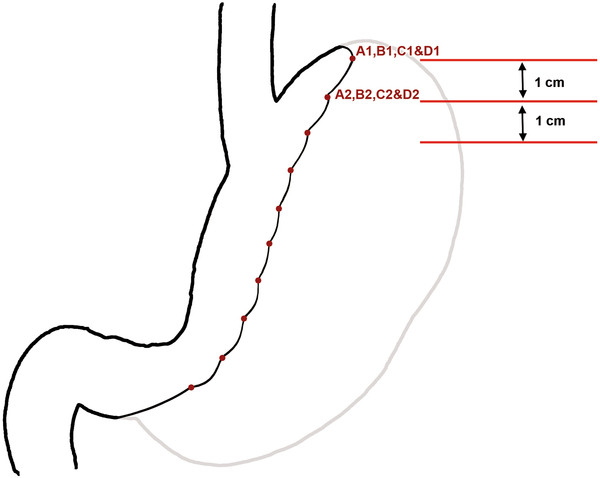
Anterior view after plication preserving His Angle.

The aim of procedure was restriction of or ideally packing as much space of stomach as possible via folds (plications) from its own wall. During first 6 years of experience, one-row plication was performed, followed by 6 years of two-row plication (Figure 2). It was done by invagination of three sections of gastric wall from the perimeter of greater curvature. As illustrated schematically in Figure 3, in order to have three sections (AB, BC and CD), it was obviously needed to have four separation points of A, B, C and D (which represent the locations of suture bites). There would be four bites at each transverse level; two (A and B) in anterior and two (C and D) in posterior gastric wall. These points were repeated at many levels (1, 2, 3 …, n) from top at the fundus to bottom at prepyloric area of stomach. At each level, bites A and D were about 1 cm (but increasing proportional to the diameter of respective stomach section) away from right aspect of the stomach and B and C were about 1 cm away from the greater curvature. Points A and D in all levels together comprise outer or superficial suture row while Bs and Cs make the deep or inner suture row (Figure 4). These two rows would not be practically separate at the end because pulling the thread passed across the four points will approximate all the points together at the left side of stomach (Figure 5). The thread was never to be cut during the procedure and all the bites were continuous. For the first level of bites, bite one (A1) was inserted almost 2cm above His angle below the attaching part of fundus to the diaphragm. Next bite (B1) was taken at anterior and near the greater curvature; then the posterior ones C1 and D1 were taken from posterior wall respectively mirror to B1 and A1 bites. Pulling the thread resulted in inward plication of greater curvature composed of three folds of AB, BC and CD. The three folds were almost the same size and had almost 2 cm bulk of gastric wall. Next level was 1 cm below the first one and suturing order was started again from A1 not D1. Continuing this procedure, level by level, ended in plication of three longitudinal folds of gastric wall into stomach. 00 prolen or nylon was used. Sutures were seromuscular, so that it was far away gastric acid. To assure the best site for suture intraluminal guide (No. 36) was used primarily. The gastric volume was calculated in passive state in operating room at the end of operation in first ten cases. The anatomic volume was measured by transient occlusion of pylorus by an atraumatic grasper and infusion of liquid to stomach freely and without any forced pressure (so that it was equal to atmosphere pressure) using red nasogastric tube (NGT) until we can see the level of liquid at the mouth. The calculated volume minus the volume of retained liquid in the esophageal part of NGT was taken as the real volume of stomach.

### Postop

In the very first cases (about 10) postoperative endoscopy was performed to find out position of inverted fold. But no guide or endoscopy was applied routinely later on.

Intravascular infusion of liquid (4-5 liters of dextrose half saline daily) was advised after operation to decrease the risk of DVT and increase the potential of mobilization. Patients did not have Foley catheter or NGT after operation and they were mobilized after about 4 hours of operation. ICU admission was not routinely advised. Patients were discharged after 24 hours of operation in the first 100 cases but the plan changed to 3 days observation with high amount of IV infusion due to very low intake of water. The average time of follow up was 5 years (1 month to 12 years).

During first 6 postoperative weeks no solid food which was soft-liquid (2Weeks), semi-liquid (2Weeks) and semi-solid food (2Weeks) were advised until the patient could be able to eat normal meal (Table 2).

**Table 2:** Plan of diet after LGP

**TIME/DIET**	**LIQUID**	**SEMI LIQUID**	**SOLID**
**HOSPITAL**	4-5 LITERS SERUM THERAPY	-	-
**1TH WEEK**	1 LITER (MILK, JUICE, WATER, SYRUP)	-	-
**2TH WEEK**	2 LITER(MILK, JUICE, WATER, SYRUP)	-	-
**3-4**^**TH**^**WEEKS**	400CC SOUP, 800CC WATER	SLICED FRUIT, YAGURT	-
**5-6**^**TH**^**WEEKS**	800CC WATER	400CC SOUP AND MEAT, YAGURT	FRUIT
**1.5-3 MONTHS**	800CC WATER	YAGURT 100CC	BREAD 50 gr, CHEESE, MEAT 50 gr
**4-6 MONTHS**	800CC WATER	YAGURT 200CC	BREAD 100 gr, CHEESE, MEAT 100 gr

The volume of prescribed liquid intake was 2 liters per day orally or intravascular infusion after hospital discharge. It should be noted that the patients are unable to take more than 1 liter of liquid in first week due to edema which subsides afterwards.

Supplemental vitamin and especial trace elements were advised. Patients took heparin (prophylactic), pantazol, antibiotics, vitamin C and B complex, apotel, pethidine, metoclopramide and ondansetron during first 2 days. Then milk of Magnesium, syrup of multivitamin and syrup of acetaminophen for the first month used and followed by routine capsules of vitamins.

We also assessed the amount of fluid the subjects were able to take at early postop and at first 6 and 24 months intervals. The measure was based on patient’s potential of painless eating. EWL was recorded regularly after surgery.

Control sonography was done to assess the liver condition in one year time. All patients were followed at regular sessions recording weight changes and investigated for any complications. Functional gastric volume and its changes were recorded periodically. Functional gastric volume was defined as maximum amount of intake above which it induces sharp pain or vomiting which was initial preventive mechanism from excessive oral intake.

The rate of gastric volume expansion was compared between one-row and two-row plications. It was also evaluated by endoscopy and contrast study for any unwanted expansion event.

All of patients were on diet and exercise as the order. The main point of their diet was to decrease glucose and fat as much as possible. The prescribed volume of oral intake was dependent to the type of food ingredients. This should be considered because the functional gastric volume was associated with food ingredients such as proteins, carbohydrates or water due to different levels of peristalsis induced to gastric muscle.

### Diet

The diet designed to contain 800 kilo calorie per day in first 6 months after surgery. After 24 hours of operation soft liquids such as milk (100cc), syrup as solution of sugar in water (200cc), fruit juice (300cc) and water (400cc) started with up to 25 cc each 15 minutes during the wakefulness time (1 liter) (first plan) . After 1 week of operation the volume of the same soft liquids increased up to 50 cc each time (2 liters daily) for one week (second plan) due to reduction in edema. During the third and fourth weeks syrup was changed to the liquid extract of boiled meat and vegetables, milk changed to soft yogurt and juice to slice fruits (apple, orange) with the same volume (Third plan). The aim of third plan was to stop any sugar intake and start cooked foods supplying mainly proteins and vitamins. Fourth plan of diet was about 2 weeks and included normal yogurt, whole dish of boiled meat and vegetable, normal fruits and 1 liter of water. The strategy of ingesting 50 cc each time was continued for at least 6 months. The fifth and sixth plans of diet were about 1.5 and 3 months and included bread, cooked meat and salad. Exercise in the form of walking for 1 hour each day was the advised plan from the second month of operation. Supplemental therapy began immediately in the form of spray and syrup at first and continued after 1 month by capsules (Table 2).

## Results

In this prospective case series study 800 cases were included. The mean age of patients was 27.5 years old (12-65). Nine patients less than 18 years old (adolescents) also were included in the study whose indication for surgery was high speed of weight gain and risk of super obesity. For adolescents, Excess weight loss (EWL) was generally the same as adults. 9 adolescent patients had 54% EWL after 6 months and 62% after 12 months. In all the ratio of female to male was 650 to 150 (81% to 19%). Mean BMI of patients was 42.1 (35-59). The mean EWL was 20% (13% to 40%) after one month (779 cases), 35% (20% to 60%) after 2 months (745 cases), 45% (25% to 75%) after 3 months (711 cases), 60% (28% to 100%) after 6 months (615 cases), 67% (35% to 100%) after 12 months (491 cases), 70% (40% to 100%) after 24 months (356 cases), 66% (35% to 100%) after 3 years (251 cases), 62% (30% to 100%) after 4 years (176 cases) and 55% (28% to 100%) after 5 years (134 cases) following surgery. The average time of follow up was 5 years (one month to 12 years). 134 (16.7%) cases were lost to follow-up in long term and partially included in EWL results (Table 3).

**Table 3:** Excess weight loss after LGP

**PLICATION**	**1 MON**	**2 MON**	**3 MON**	**6 MON**	**1 YEAR**	**2 YEAR**	**3 YEAR**	**4 YEAR**	**5 YEAR**	**10 YEAR**
**EWL**	20%	35%	45%	60%	67%	70%	66%	62%	55%	42%
**PATIENTS**	779	745	711	615	491	356	251	176	134	35

*EWL*: excessive weight loss.

The technique of laparoscopic gastric plication (LGP) was performed in all cases including those with large fatty liver, hiatal hernia and adhesions from previous operation. Sonography showed gallstone in 52 cases (6.5%) and cholecystectomy was performed at the same time. History of cholecystectomy was positive in 21 cases (2.6%).

Fatty liver was reported in 85% of cases (682 cases) by sonography in different grades including: 421 cases (52%) grade G1, 154 (19%) cases G2 and 107 cases (13%) G3. After one year of LGP report of fatty liver by check-up sonography was as below: 211 out of 242 cases (87%) of G1 recovered completely, 45 cases out of 91 (49%) of G2 recovered completely and 27 (29%) of them changed into G1,35 out of 102 cases (34%) of G3 recovered completely and 48 cases down staged (47%).

**Figure 6:**
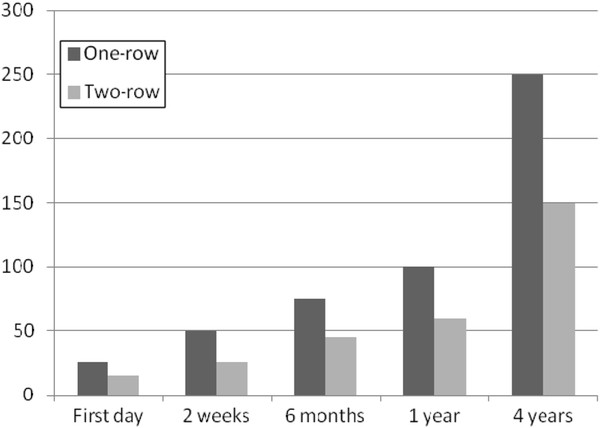
Trend of functional intraluminal space changes in 2 methods of LGP.

The average anatomic volume of stomach in the operating room was 100 and 50 cc in one- and tow-row plication respectively. But The functional volume of stomach in one- and two-row LGP respectively was about 25 and 15cc at first, 50 and 25cc after 2 weeks, 75 and 45cc after6 months, 100 and 60cc after 1 year and 250 and 150cc after 4 years (Figure 6). The functional volume was highly related to the kind of food. If the patient was eating something rich of proteins the amount of functional intake was considerably less compared with the time something with high carbohydrate ingredients was taken. In some especial form of diet such as plain water or sweet water etc. the functional restriction did not happen until the anatomic volume was reached. The appetite of patients decreased after operation due to total gastric volume restriction. They described this feeling as like the condition after eating more than usual with complete fullness of stomach.

**Figure 7:**
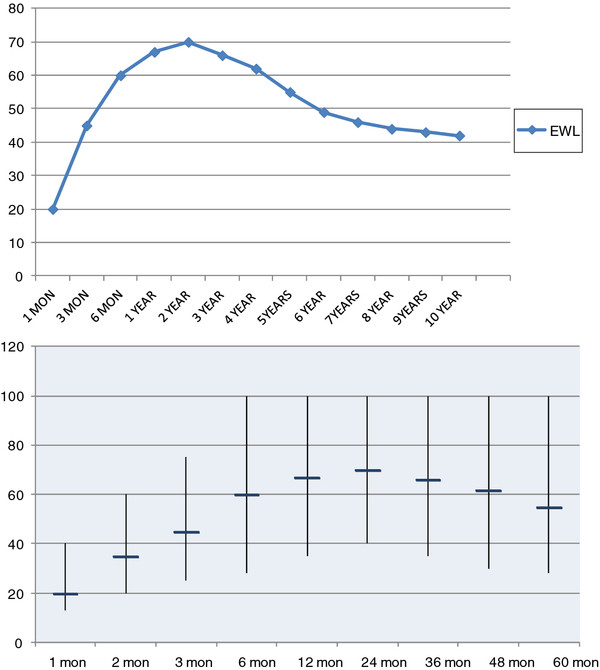
EWL after LGP, A Mean Percentages of EWL from baseline amount during 5 years of follow up; B Mean Percentages of EWL from baseline amount during 5 years of follow up and their variance in cases and its range as vertical lines.

The weight loss curve had prominent slope during first 6 months but for next 18 months showed decreased rate (Figure 7).

Mild to moderate weakness during first 3 weeks was common. Vomiting and nausea was seen in all of cases for at least 4 hours and the longest time was 24 days (average time was 2.1 days) which resolved spontaneously. Epigastric pain was seen in 35% of cases for 48 hours which relieved quickly by antacids. Temporarily during the first Postop week, reflux was seen in 16% (128 out of 800) of cases without any preop history of reflux symptoms. It changed to less than 2% after 3 months concurrently with about 45% EWL.

16 patients out of 615 had problems after 6 months which only one of them required reoperation to undo the plication due to long term vomiting secondary to adhesion of liver to the His angle. Others including 2 patients with gastritis and 13 with persistent reflux were controlled by medical therapy (Table 4).

**Table 4:** Postoperative problems after LGP

**POSTOPERATIVE PROBLEM**	**DETAIL**
**VOMITING**	common, mean 2 days postop
**REFLUX**	2% after 3 months
**EPIGASTRIC PAIN**	35%, after 48 hours,

The price of hospitalization and instruments used in gastric plication was 2000$, while gastric bypass would cost 4500$ and sleeve or banding 4000$ in Laleh Private Hospital [28].

### Complications

The rate of unrelated complications was 0.6% (5 cases out of 800). In two cases, non-obstructive jaundice appeared for more than 2 weeks after operation which resolved spontaneously. Liver enzymes were very high. The etiology was drug induced hepatitis.

Symptomatic hypocalcemia was seen in one patient secondary to lack of intake. She had hypercalciuria in her past medical history and since she got enough calcium supplements, became asymptomatic. Aspiration pneumonia occurred in one subject who underwent 2 weeks postoperative treatment. Mild bleeding due to anticoagulation therapy was seen in one case which stopped by conservative management and 2 units of fresh blood transfusion. Although dissection of greater curvature was with the risk of bleeding but in our cases blood transfusion was needed only once.

Postoperative technical complications were seen in 8 cases out of 800 (1%). Micro perforation occurred in three cases; the first one occurred at the site of gastric holding by grasper at prepyloric area which was closed by simple suture without any change in plication via laparotomy; one case at the site of needle insertion at upper end of plication due to increased intraluminal pressure and its dilation in one point which was treated by simple suture by laparoscopy; and the last one due to fundus sliding outside of suture row and blowout of dilated displaced fundus. Treatment of this case was by laparotomy, undoing the suture line and drain insertion. During follow up it took about 2 weeks for fistula to evolve and closure of fistula completed after 45 days and drains were taken out afterwards.

Intrahepatic hematoma due to fan retractor manipulation predisposed intracapsular liver abscess formation after 6 months in one case. The hematomas did not occur again due to using the new question mark liver retractor.

Postoperative obstruction presented by continuous vomiting was seen in three cases due to displacement of released fundus outside the suture line and extra-expansion. But instead of dilation at needle insertion point or blowout, the displaced folds stretched the string, tightening the rest of the knots especially the last one near pylorus. The stomach outflow kinked and produced an obstruction. The management was via laparoscopy. The suture line was undone and replication performed. The last tie close to the pylorus was done relatively looser than before.

In another case due to unusual adhesion between fundus and traumatized liver, permanent vomiting and discomfort was seen. Actually in this case laparoscopic reoperation 8 months later resolved the problem. In this surgery the adhesion was released and plication was undone.

Undoing of LGP in first case by cut of thread and separation of folds performed with limited adhesions. In second one adhesion between folds was really strong and separation was hard (Table 5).

**Table 5:** Postoperative complications after LGP

**REOPERATION**	**DETAIL**	**TECHNICAL INDIPENDENT**	**DETAIL**
**PERFORATION**	3 CASES	HEPATITIS	HALOTAN INDUCED, 2 CASE
**OBSTRUCTION**	3 CASES	HYPOCALCEMIA	RARE CASE REPORT
**PERMANENT VOMITING**	1 CASE	ASPIRATION	1 CASE
**INTRACAPSULAR ABSCESS**	1 CASE	BLEEDING	1 CASE
**TOTAL**	8 CASES (1%)	TOTAL	5 CASES (0.6%)

**Figure 8:**
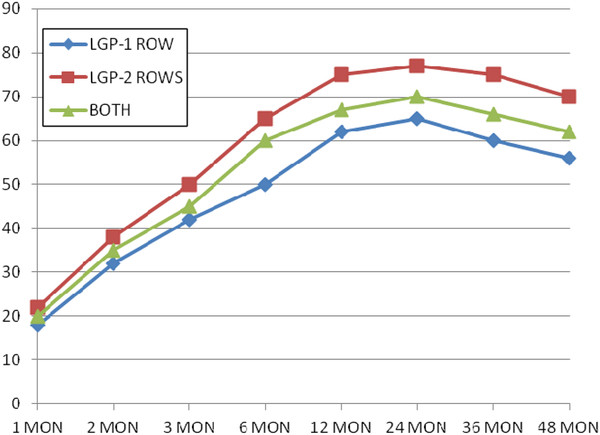
Comparing Excess Weight Loss between one- and two-row LGP.

156 and 644 cases underwent one- and two-row plication respectively. All of complications were seen in former technique except two obstruction cases in the latter (0.3%). Comparing EWL showed it was the same at first but higher at long term due to less anatomic volume and prominent functional restrictive effect in two-row technique. (50% and 65% after 6 months, 62% and 75% after 12 months, 65% and 77% after 2 years, 60% and 75% after 3 years and 56% and 70% after 4 years in one- and two-row respectively) (Figure 8).

**Figure 9:**
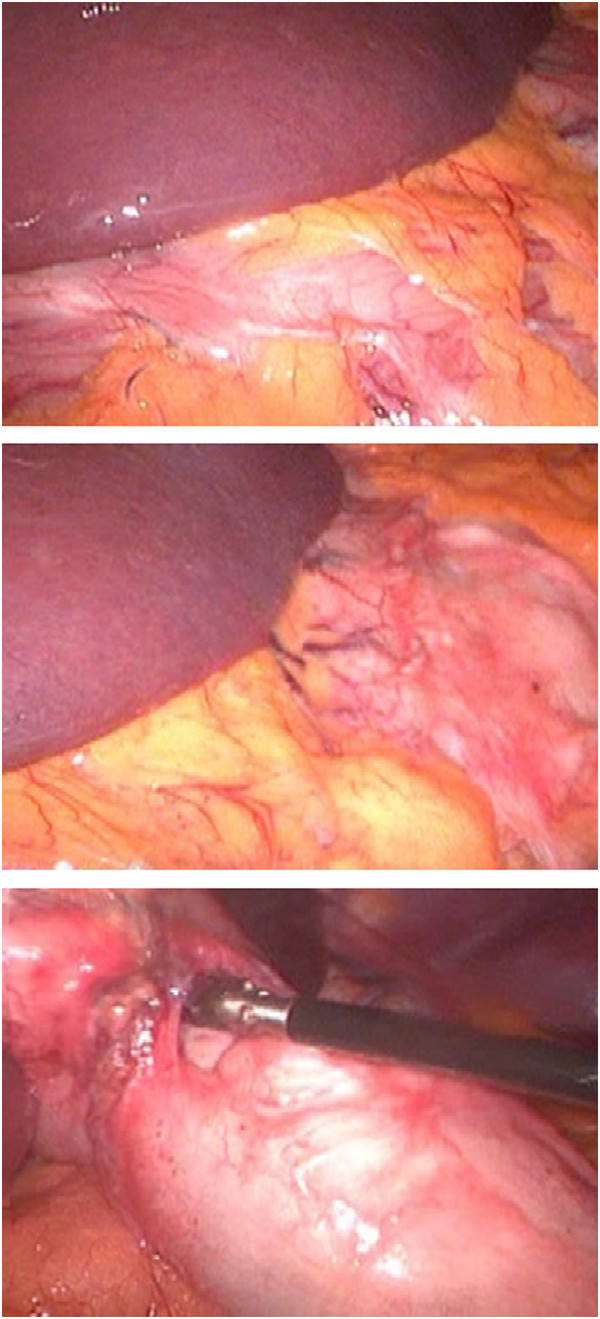
Plicated stomach after 3 years.

Reoperation due to regain (32), failure (6) or other reasons (gallstone 12, appendicitis 3) was done in 53 cases and plication rechecked. These cases showed unchanged suture line but little expansion of the stomach (Figure 9). Fibrotic bands around plication had reinforced it. In 25 out of 38 cases of regain or failure, outside displacement of plicated fold was seen (65%).

The rate of late (after 1 month of operation) postoperative complication was zero. Some comorbidities was present before surgery in 18% of cases including 11% diabetes, 5% knee or low back pain, 4% hypertriglyceridemia, 1% hypertension, and 0.5% sleep apnea. Six months and one year after operation, respectively 70% and 95% of diabetic cases changed into non diabetics (40% after 2 months) and the remaining needed to taper their medical therapy; 80% and 100% knee or low back pain, 40% and 70% of hypertriglyceridemia, 50% and 80% of hypertension and all of sleep apnea cases recovered from diseases. 2 cases out of 55 regain patients complained of diabetes again (Table 6).

**Table 6:** Comorbidities in LGP cases and the recovery percentage

**COMORBIDITY**	**Frequency**	**% OF RECOVERY AFTER 6 MON**	**% OF RECOVERY AFTER 1 YEAR**
**DIABET**	11%	70%	95%
**HYPERTENSION**	1%	50%	80%
**KNEE OR LOW BACK PAIN**	5%	80%	100%
**HYPERTRIGLYCERIDEMIA**	4%	40%	70%
**SLEEP APNEA**	0.5%	100%	100%
**TOTAL**	18%	35%	67%

The mean time of operation was 72 (49–152) minutes; all were discharged after an average time of 72 hours (24 hours to 45 days). It was 100 minutes when dissection of greater curvature was done by coagulation and suture and 67 minutes when it was done by LigaSure^TM^ or Ultracision.

### Regain and failure

In experience there was about 31% regain after up to 8 years of effective time (4 year period) of operation (55 out of 176), which was 5.5% up to 4 years after operation (26 out of 490) and 42% after 10 years (15 out of 35). Cases with less than 30% EWL after 4 years were included in regain group. We put 6 failure cases with less than 30% EWL during first 6 months in it as well.

The main regain and failure group was cases with wrong selection of technique, mainly males without good motivation and co-working (31 regain and 6 failure cases). The plan for this group was malabsorptive operation that has done in 20 cases with excellent EWL (78% EWL after 1 year). The second group was cases with good result secondary to plication, but due to new conditions (like marriage, pregnancy and psychological disease secondary to familial problems like divorce, death of relatives and so on) lifestyle of patient has changed again. Patients with temporary change of conditions like pregnancy can be managed by replication or gastric bypass. But if the new condition was permanent, the method of choice was malabsorptive. In 11 out of 18 such cases we did replication, gastric bypass in 2 and malabsorptive in 5. The mean EWL after 6 months of replication in these cases was 44% and after 1 year it was 51% with mean follow up time of 24 months (55%) (Table 7).

**Table 7:** Regain or failure after up to 8 years of effective period of LGP (4 years)

**REGAIN**	**NUMBER**	**NEW PLAN**	**OUTCOME**
**WRONG SELECTION**	31 regain	MALABSORPTIVE	20 CASES,
	6 failure		78% EWL/ 1 YEAR
**TEMPORARY NEW CONDITION**	13	11 REPLICATION	11 CASES LGP,
		2 GASTRIC BYPASS	55% EWL/ 2 YEAR
**PERMANENT NEW CONDITION**	5	MALABSORPTIVE	5 CASES
**TOTAL**	55/176 (31%)	68% MAL,	38 CASES,
		32% REPLICATION	69% REOP

## Discussion

LGP as a new restrictive method for treatment of morbid obesity has shown acceptable results during its evolution since 12 years ago. The complications were not more than other methods; and costs were far less than any other bariatric surgery. The final result of weight loss was almost the same as other restrictive techniques [29]. However, the wide range of weight loss resulted from different methods of restrictive bariatric surgery means the main reason of weight loss is not just a specific technique; but also could be mostly the patient’s motivation to keep appropriate lifestyle [11,30].

### Evolution of technique

**Figure 10:**
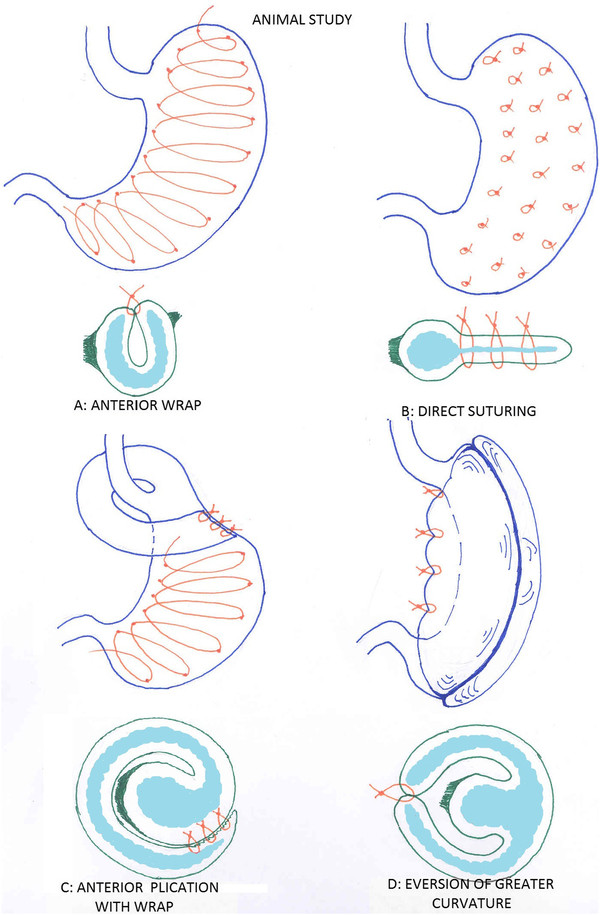
Four plans of restrictive method in animal study.

**Figure 11:**
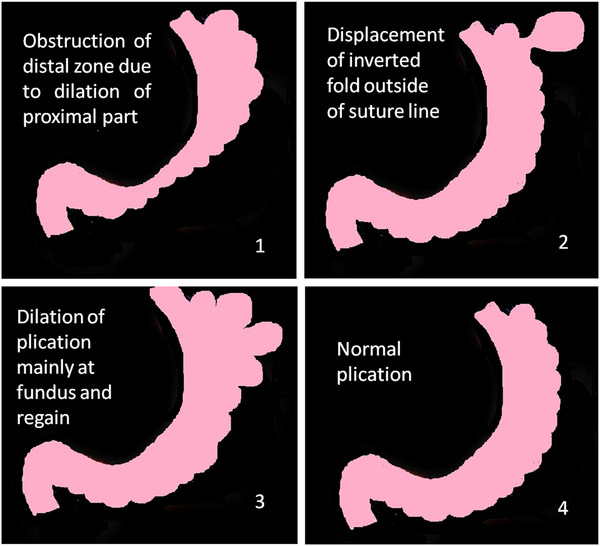
Complication of one row plication.

**Figure 12:**
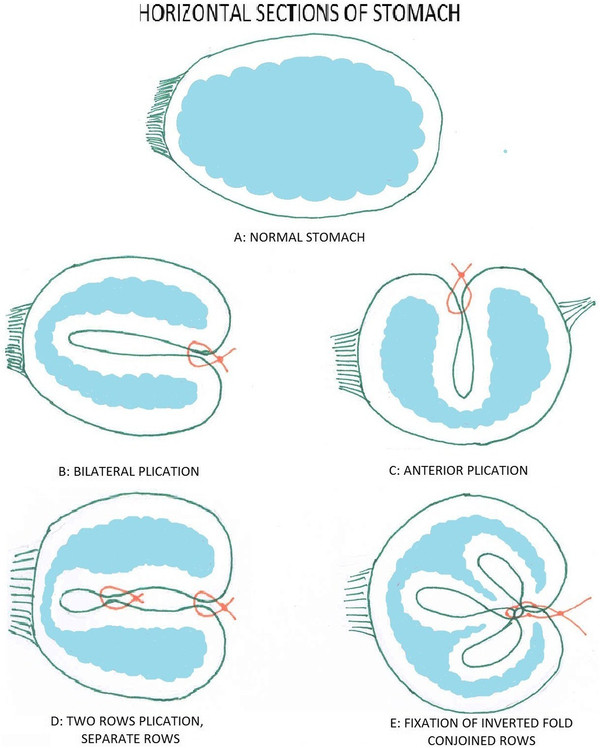
Different methods of plication.

Four plans of restrictive method were studied in animal lab (Figure 10). The final version was taken as safest and most effective method to be performed on volunteer cases in year 2000. Three further modifications applied on cases resulted in acceptable one-row plication. But after occurrence of few complications mainly displaced bulging of some plications out of suture line (Figure 11), it had to be more optimized to the two-row plication (Figure 12).

Presentation of early 4 year results of LGP by author [29] increased the interest of famous bariatric surgeons in the world to begin this new bariatric method with acceptable same results [31-33].

Outcome of plicated stomach is well known after about 12 years experience. All technical complications occurred during first week after operation. If the patient could pass first postoperative week without any problem, there was not any risk of complication later on. As the wall of stomach was elastic and intake of food could make extension, increase of intraluminal space occurred gradually. During the first 14 days after operation metabolism of fat and sugar has been probably changed in the liver. The weakness during first 3 weeks was probably due to lack of enough fat and glucose intake and the interval needed for physiologic adaptation in the body to supply glucose by lipolysis in the liver.

The average volume of painless drinking after one-row plication was about 50 cc each time. The main reason was contraction of gastric muscles and also painful peristaltic movement in higher volumes (functional restrictive effect). The volume of stomach in two-row plication was 50 cc (anatomic restriction) but the role of functional restriction in this method was more prominent.

If patient took more than 25 cc each time epigastric pain or esophageal reflux would occur (especially during first 6 months). These were two inhibitory mechanisms preventing any change in volume intake. If volume intake was in permitted dose, reflux or pain would not appear.

Functional restriction was due to adhesion of two-row sutures together in which any form of muscular movement could be painful. If two-row sutures get separate, there would be two problems: the inverted fold would be more mobile and risk of removal or displacement and vomiting is higher, and the functional restriction would decrease.

The feeling of postoperative gastric fullness was the possible reason for nausea in all cases, which would be corrected after adaptation.

Temporary postop reflux without any preop history was probably due to high intraluminal pressure and mucosal edema caused by plication. This was observed especially in cases with incompetent lower esophageal sphincter which is seen commonly in morbid obesity. The rate of reflux dropped down due to subsidence of edema after one week.

As the length of string was fixed and the process of gastric extension was gradual, it showed by endoscopy that the string was routinely transposed into the lumen of stomach and removed from the body by defecation after 4 years of operation. Based on endoscopy and mucosal biopsy and chyme analysis in randomized cases and according to reoperation (due to gallstone or appendicitis) findings for regain, plicated fold showed shrinkage due to muscular atrophy, but the mucosa and its circulation was normal because it was in contact to food; so that, its enzymatic, hormonal and mucosal secretion was normal. Shrinkage of plicated fold and extension of elastic gastric wall were two main factors for limited effective time of plication (4 years).

Probability of regain after about 4 years of gastric plication is a fact. If we select the patients correctly and they successfully change their lifestyle, after effective time of operation (4 years), they could have a good new condition (normal weight, acceptable psychological mood, new lifestyle) that can prohibit any form of regain. The rate of 42% regain after 10 years in first 35 cases of LGP was due to learning curve (increase in surgical expertise), one-row method, far away bites from lesser curvature. It needs more cases and more time especially in two-row LGP cases to assess meaningful long term result.

Replication was easier than plication, because release of greater curvature had been done before, adhesions of liver or omentum to stomach were loose and re-suturing over dilated plicated stomach was something like the second row suturing in plication method.

Long term results depend mainly on the extent of motivation to keep on corrected lifestyle. Restrictive effect of surgery takes until 4 years and then enlargement of stomach up to 250 cc is enough to induce regain. But in experience 5.5% of cases got regain during first 4 years (effective period) and 31% up to 8 years after effective period of LGP (12 years) and the remaining cases preserve their new lifestyle for long term period. Unfortunately it is not for all life and the percentage rate of regain increased gradually. As the main group of cases were young females and they were interested in plication due to very low complication rate, we think the main factor for increasing motivation of these patients was social factors like marriage; so that after one decade of operation these social factors have been solved and patients’ motivations has decreased. Consideration of morbid obesity as a disease and trying to control it was not sufficient motivation for them.

### The advantages of LGP

As the effects of all restrictive methods are almost the same, the best method is the one with the least risk of complication. Gastric plication has the least rate of reoperation which is due to 1% technical (in the first week) and less than 0.2% late complications. It is noteworthy in comparison to other restrictive methods like gastric banding or sleeve gastrectomy. There is about 10% to 20% of emergency band removal (happening in wide range of time after surgery) in gastric banding operation because of balloon leakage, band erosion/migration, deep infection and reaction to foreign body [34]. Reoperation to remove balloon is the most important disadvantage of this technique [27,35-38]. In vertical banded gastroplasty (VBG) the risk of reflux, leak, blow out of stomach, regain and foreign body reaction would be highly observed [30]. Complications secondary to sleeve gastrectomy including leakage, disruption and malabsorption is about 10% [36,37,39,40]. Effective time of all restrictive methods is limited to 3 to 4 years, so risk of regain is the problem of all kinds of bariatric surgeries.

Especial hormones of stomach (Ghrelin, Leptin etc.) make an appetite balance in normal condition. Although the effect of ghrelin on appetite is remarkable, balance between gastric hormones and appetite was not changed after sleeve gastrectomy [13-17]. Losing appetite is related to decreased intraluminal space and high intraluminal pressure. This mechanism is more noticeable in gastric plication. When intraluminal space increases during time, appetite increases as well regardless of surgical technique used.

To sum it up, advantages of LGP over other restrictive methods are as follows. First, the patient is independent postoperatively with easy follow up; which means the patient is free from any obligatory post-operative procedures e.g. balloon size regulation in gastric banding. No foreign body reaction is the next advantage of LGP as only two or three prolen or nylon threads are used with no use of mesh or band. Moreover, less cost due to no need for stapler or band and short period of postoperative hospitalization are very important factors for patients. Less complication such as leakage, infection or erosion should be noted since this method is the most conservative procedure among other bariatric surgeries with no resection or anastomosis.

Psychological situation in these patients is satisfying since they are independent to surgeon after the surgery and have no fear of any complications of foreign bodies. It is interesting that 4 years after gastric plication patients will have no sign of surgery in their stomach, the string will pass through GI tract, muscular layer of inverted folds will be hypotrophied and actually weight loss will occur without any change or sequel in the body.

If needed, this method is reversible during first 6 weeks just by cutting the suture lines and easily releasing the mild adhesions. Reversion of LGP later is difficult and it needs tense fibrotic tissue dissection. It can be followed by adding malabsorptive method as a second stage operation in cases with insufficient weight loss without any change to previous surgery. EWL in this technique is the same as other restrictive surgeries (Table 8).

**Table 8:** Advantages of LGP

**ADVANTAGES**	**DETAIL**
**LACK OF FOREIGN BODY**	PROLEN
**LOW MORBIDITY**	VOMITING
**COST EFFECTIVE**	ANY STAPLER
**LOW COMPLICATION**	1%
**INDIPENDENT**	PSYCHOLOGICALLY
**REVERSIBLE**	CUT OF THREAD
**LACK OF APPETITE**	LACK OF SPACE
**FUNCTIONAL RESTRICTIVE EFFECT**	PAINFUL PERISTALTISM

Comparing this technique with using stapler and resection of greater curvature, LGP is less invasive and more conservative with reversible potency and less risk of complications such as leakage.

Making both wrap and plication to decrease the volume of stomach predisposes higher risk of suture pressure and possibility of leakage. Anterior plication without release of greater curvature is less invasive but has more risk for weight regain.

If patient is poorly cooperative and less likely to change lifestyle in the future, restrictive operation is not a good option and patient needs malabsorptive bariatric surgery. Although risk of morbidity and mortality in malabsorptive methods are higher than restrictive, this is a good choice in this group [14,41-43].

In duodenal switch (DS) there is a significant mal-absorptive component, so the patient must be closely monitored for nutritional deficiencies. These patients are at greater risk of chronic diarrhea, more foul smelling stools and flatus. In gastric bypass because it includes resection, anastomosis and malabsorption, the risk of mortality and morbidity are 1% and up to 15% respectively [44].

Although the risk of mortality and morbidity in bariatric surgery is high (less than 1% mortality and 10% morbidity), most patients accept the risk because of their miserable and limited life secondary to morbid obesity.

A special diet after restrictive operation with high speed weight loss has made the question about composition of lost tissue [45]. Is all of it fatty tissue? Or water and protein has lost secondary to this form of high speed weight loss.

The mechanism of high catabolism of fatty tissue after operation in comparison to traditional diet is not well known but enzymatic and hormonal change in the body could be the main factor. More study in this field can be helpful to find new strategies for treatment of morbid obesity in the future.

## Conclusion

Laparoscopic gastric plication is as effective as other restrictive methods to lose weight. Its advantages include: easy follow up, no foreign body, much less cost, low complications (0.6%), low reoperation (1%), only 31% regain up to 12 years follow up, easy plan for regain group, psychological advantage due to independency to surgeon and sense of normal physiology and anatomy without any resection. This method is reversible if needed and also does not prohibit complementary malabsorptive methods (2 stage operation) in cases with insufficient weight loss.

## Abbreviations

RM, Restrictive method; LGP, Laparoscopic gastric plication; EWL, Excess weight loss; RGEA, Right gastroepiploic artery; NGT, Nasogastric tube.

## Competing interests

The authors declare that they have no competing interest.

## Authors’ contributions

MT has evolved the surgical technique and participated in conception and design of study. He was responsible for obtaining funding for study and final approval. SMKM was responsible for critical revision of study manuscript and participated in scientific writing. AT participated in provision of study material and collection and assembly of raw data. HV was responsible for analysis and interpretation of data, participated in collection of data and writing the manuscript draft. He is corresponding to letter. All authors declare that they have read and accepted the final manuscript to be submitted.

## Authors’ information

Mohammad Talebpour got his general surgery degree from Tehran University of Medical Sciences (TUMS). His subspecialty is advanced laparoscopy from Ninewells Hospital, UK. He is associate prof of TUMS, Head of laparoscopic ward, Sina Hospital, Tehran, Iran.

SMKM is an MD/MPH graduate from Tehran University of Medical Sciences (TUMS). He has been involved in endocrinological research and related fields since 2009 in Endocrinology and Metabolism Research Center (EMRC) in Iran. He has been recently started cooperation with Laparoscopic Surgery Ward of Sina Hospital. Atieh Talebpour is an MD graduate from Tehran University of Medical Sciences (TUMS). She has been involved in surgical research in Laparoscopic Surgery Ward of Sina Hospital.

Hamed Vahidi is an MD/MPH graduate from Tehran University of Medical Sciences (TUMS). He has been involved in surgical research in Laparoscopic Surgery Ward of Sina Hospital. He is the second student of general examination of medical science in Iran. His research experience was with Endocrinology and Metabolism Research Center (EMRC) and Pharmacologic Research Center in Iran.

## References

[B1] Drewnowski A Popkin BM The nutrition transition: new trends in the global diet Nutr Rev 1997 55 31 43 9155216 10.1111/j.1753-4887.1997.tb01593.x

[B2] Popkin BM The Nutrition Transition: An Overview of World Patterns of Change Nutr Rev 2004 62 7 Pt 2 S140 S143 15387480 10.1111/j.1753-4887.2004.tb00084.x

[B3] Posner BM Franz MM Quatromoni PA Gagnon DR Sytkowski PA D’AGOSTINO RB Cupples LA Secular Trends in Diet and Risk Factors for Cardiovascular Disease: The Framingham Study J Am Diet Assoc 1995 95 171 179 10.1016/S0002-8223(95)00043-7 7852683

[B4] Popkin BM The nutrition transition: an overview of world patterns of change Nutr Rev 2004 62 S140 S143 15387480 10.1111/j.1753-4887.2004.tb00084.x

[B5] Finucane MM Stevens GA Cowan MJ Danaei G Lin JK Paciorek CJ Singh GM Gutierrez HR Lu Y Bahalim AN National, regional, and global trends in body-mass index since 1980: systematic analysis of health examination surveys and epidemiological studies with 960 country-years and 9· 1 million participants Lancet 2011 12; 377 9765 557 67 10.1016/S0140-6736(10)62037-5 PMC4472365 21295846

[B6] Ruesten A Steffen A Floegel A DL A Masala G Tjonneland A Halkjaer J Palli D Wareham NJ Loos RJ Trend in obesity prevalence in European adult cohort populations during follow-up since 1996 and their predictions to 2015 PLoS One 2011 6 e27455 10.1371/journal.pone.0027455 22102897 PMC3213129

[B7] Basterra-Gortari FJ Beunza JJ Bes-Rastrollo M Toledo E Garcia-Lopez M Martinez-Gonzalez MA [Increasing trend in the prevalence of morbid obesity in Spain: from 1.8 to 6.1 per thousand in 14 years] Rev Esp Cardiol 2011 64 424 426 10.1016/j.recesp.2010.06.010 21411209

[B8] Komlos J Brabec M The trend of mean BMI values of US adults, birth cohorts 1882-1986 indicates that the obesity epidemic began earlier than hitherto thought Am J Hum Biol 2010 22 631 638 10.1002/ajhb.21055 20737610

[B9] Flegal KM Carroll MD Ogden CL Curtin LR Prevalence and trends in obesity among US adults JAMA 1999-2008 303 235 241 20071471 10.1001/jama.2009.2014

[B10] Himpens J Cadiere GB Bazi M Vouche M Cadiere B Dapri G Long-term Outcomes of Laparoscopic Adjustable Gastric Banding Arch Surg 2011 146 7 802 807 10.1001/archsurg.2011.45 21422330

[B11] Deveney CW Martindale RG Factors in selecting the optimal bariatric procedure for a specific patient and parameters by which to measure appropriate response to surgery Curr Gastroenterol Rep 2010 12 296 303 10.1007/s11894-010-0117-0 20556553

[B12] Farrell TM Haggerty SP Overby DW Kohn GP Richardson WS Fanelli RD Clinical application of laparoscopic bariatric surgery: an evidence-based review Surg Endosc 2009 23 930 949 10.1007/s00464-008-0217-1 19125308

[B13] Brody F Minimally invasive surgery for morbid obesity Cleve Clin J Med 2004 71 289 293 296-288 10.3949/ccjm.71.4.289 15117170

[B14] Garb J Welch G Zagarins S Kuhn J Romanelli J Bariatric surgery for the treatment of morbid obesity: a meta-analysis of weight loss outcomes for laparoscopic adjustable gastric banding and laparoscopic gastric bypass Obes Surg 2009 19 1447 1455 10.1007/s11695-009-9927-2 19655209

[B15] Sasse KC Ganser JH Kozar MD Watson RW Lim DC McGinley L Smith CJ Bovee V Beh J Outpatient weight loss surgery: initiating a gastric bypass and gastric banding ambulatory weight loss surgery center JSLS 2009 13 50 55 19366541 PMC3015916

[B16] Kim TH Daud A Ude AO DiGiorgi M Olivero-Rivera L Schrope B Davis D Inabnet WB Bessler M Early U.S. outcomes of laparoscopic gastric bypass versus laparoscopic adjustable silicone gastric banding for morbid obesity Surg Endosc 2006 20 202 209 10.1007/s00464-005-0243-1 16341569

[B17] Langer FB Reza Hoda MA Bohdjalian A Felberbauer FX Zacherl J Wenzl E Schindler K Luger A Ludvik B Prager G Sleeve gastrectomy and gastric banding: effects on plasma ghrelin levels Obes Surg 2005 15 1024 1029 10.1381/0960892054621125 16105401

[B18] Doherty C Maher JW Heitshusen DS Long-term data indicate a progressive loss in efficacy of adjustable silicone gastric banding for the surgical treatment of morbid obesity Surgery 2002 132 724 727 discussion 727-728 10.1067/msy.2002.127687 12407358

[B19] Jacobs PP van Uchelen FA C Bruyninckx CM Donker FJ Driessen WM Breuer C [Medium-long-term good results of vertical gastroplasty in the treatment of morbid obesity] Ned Tijdschr Geneeskd 1991 135 1445 1449 1922454

[B20] Crea N Pata G Di Betta E Greco F Casella C Vilardi A Mittempergher F Long-term results of biliopancreatic diversion with or without gastric preservation for morbid obesity Obes Surg 2011 21 139 145 10.1007/s11695-010-0333-6 21116732

[B21] Ocon Breton J Perez Naranjo S Gimeno Laborda S Benito Ruesca P Garcia Hernandez R [Effectiveness and complications of bariatric surgery in the treatment of morbid obesity] Nutr Hosp 2005 20 409 414 16335025

[B22] Doherty C Maher JW Heitshusen DS Prospective investigation of complications, reoperations, and sustained weight loss with an adjustable gastric banding device for treatment of morbid obesity J Gastrointest Surg 1998 2 102 108 10.1016/S1091-255X(98)80110-8 9841975

[B23] Juhasz-Pocsine K Rudnicki SA Archer RL Harik SI Neurologic complications of gastric bypass surgery for morbid obesity Neurology 2007 68 1843 1850 10.1212/01.wnl.0000262768.40174.33 17515548

[B24] Cariani S Nottola D Grani S Vittimberga G Lucchi A Amenta E Complications after Gastroplasty and Gastric Bypass as a Primary Operation and as a Reoperation Obes Surg 2001 11 487 490 10.1381/096089201321209396 11501361

[B25] Chevallier J-M Zinzindohoué F Douard R Blanche J-P Berta J-L Altman J-J Cugnenc P-H Complications after Laparoscopic Adjustable Gastric Banding for Morbid Obesity: Experience with 1,000 Patients over 7 Years Obes Surg 2004 14 407 414 10.1381/096089204322917954 15072664

[B26] Franco JV Ruiz PA Palermo M Gagner M A Review of Studies Comparing Three Laparoscopic Procedures in Bariatric Surgery: Sleeve Gastrectomy, Roux-en-Y Gastric Bypass and Adjustable Gastric Banding Obes Surg 2011 21 9 1458 1468 10.1007/s11695-011-0390-5 21455833

[B27] Birkmeyer NJ Dimick JB Share D Hawasli A English WJ Genaw J Finks JF Carlin AM Birkmeyer JD Hospital complication rates with bariatric surgery in Michigan JAMA 2010 304 435 442 10.1001/jama.2010.1034 20664044

[B28] Campbell J McGarry LA Shikora SA Hale BC Lee JT Weinstein MC Cost-effectiveness of laparoscopic gastric banding and bypass for morbid obesity Am J Manag Care 2010 16 e174 e187 20645663

[B29] Talebpour M Amoli BS Laparoscopic total gastric vertical plication in morbid obesity Journal of laparoendoscopic & advanced surgical techniques Part A 2007 17 793 798 10.1089/lap.2006.0128 18158812

[B30] Scozzari G Toppino M Famiglietti F Bonnet G Morino M 10-year follow-up of laparoscopic vertical banded gastroplasty: good results in selected patients Ann Surg 2010 252 831 839 10.1097/SLA.0b013e3181fd35b0 21037439

[B31] Ramos A Galvao Neto M Galvao M Evangelista LF Campos JM Ferraz A Laparoscopic greater curvature plication: initial results of an alternative restrictive bariatric procedure Obes Surg 2010 20 913 918 10.1007/s11695-010-0132-0 20407932

[B32] Pujol Gebelli J Garcia Ruiz de Gordejuela A Casajoana Badia A Secanella Medayo L Vicens Morton A Masdevall Noguera C Laparoscopic Gastric Plication: a new surgery for the treatment of morbid obesity Cir Esp 2011 89 6 356 361 10.1016/j.ciresp.2011.02.005 21481852

[B33] Brethauer SA Harris JL Kroh M Schauer PR Laparoscopic gastric plication for treatment of severe obesity Surg Obes Relat Dis 2011 7 15 22 10.1016/j.soard.2010.09.023 21144804

[B34] Muller MK Wenger C Schiesser M Clavien PA Weber M Quality of life after bariatric surgery--a comparative study of laparoscopic banding vs. bypass Obes Surg 2008 18 1551 1557 10.1007/s11695-008-9522-y 18461420

[B35] Campos GM Rabl C Roll GR Peeva S Prado K Smith J Vittinghoff E Better weight loss, resolution of diabetes, and quality of life for laparoscopic gastric bypass vs banding: results of a 2-cohort pair-matched study Arch Surg 2011 146 149 155 10.1001/archsurg.2010.316 21339424

[B36] Abbatini F Rizzello M Casella G Alessandri G Capoccia D Leonetti F Basso N Long-term effects of laparoscopic sleeve gastrectomy, gastric bypass, and adjustable gastric banding on type 2 diabetes Surg Endosc 2010 24 1005 1010 10.1007/s00464-009-0715-9 19866235

[B37] Boza C Gamboa C Awruch D Perez G Escalona A Ibanez L Laparoscopic Roux-en-Y gastric bypass versus laparoscopic adjustable gastric banding: five years of follow-up Surg Obes Relat Dis 2010 6 470 475 10.1016/j.soard.2010.02.045 20702146

[B38] Gutschow CA Collet P Prenzel K Holscher AH Schneider PM Long-term results and gastroesophageal reflux in a series of laparoscopic adjustable gastric banding J Gastrointest Surg 2005 9 941 948 10.1016/j.gassur.2005.02.001 16137589

[B39] D’Hondt M Vanneste S Pottel H Devriendt D Van Rooy F Vansteenkiste F Laparoscopic sleeve gastrectomy as a single-stage procedure for the treatment of morbid obesity and the resulting quality of life, resolution of comorbidities, food tolerance, and 6-year weight loss Surg Endosc 2011 25 8 2498 2504 10.1007/s00464-011-1572-x 21359900

[B40] Omana JJ Nguyen SQ Herron D Kini S Comparison of comorbidity resolution and improvement between laparoscopic sleeve gastrectomy and laparoscopic adjustable gastric banding Surg Endosc 2010 24 2513 2517 10.1007/s00464-010-0995-0 20339873

[B41] Mognol P Chosidow D Marmuse JP Laparoscopic gastric bypass versus laparoscopic adjustable gastric banding in the super-obese: a comparative study of 290 patients Obes Surg 2005 15 76 81 10.1381/0960892052993486 15760503

[B42] Simpfendorfer CH Szomstein S Rosenthal R Laparoscopic gastric bypass for refractory morbid obesity Surg Clin North Am 2005 85 119 127 x 10.1016/j.suc.2004.10.001 15619533

[B43] Ballantyne GH Wasielewski A Saunders JK The surgical treatment of type II diabetes mellitus: changes in HOMA Insulin resistance in the first year following laparoscopic Roux-en-Y gastric bypass (LRYGB) and laparoscopic adjustable gastric banding (LAGB) Obes Surg 2009 19 1297 1303 10.1007/s11695-009-9870-2 19629603

[B44] Pontiroli AE Morabito A Long-term prevention of mortality in morbid obesity through bariatric surgery. a systematic review and meta-analysis of trials performed with gastric banding and gastric bypass Ann Surg 2011 253 484 487 10.1097/SLA.0b013e31820d98cb 21245741

[B45] Strain GW Gagner M Pomp A Dakin G Inabnet WB Hsieh J Heacock L Christos P Comparison of weight loss and body composition changes with four surgical procedures Surg Obes Relat Dis 2009 5 582 587 10.1016/j.soard.2009.04.001 19560983

